# Desirable treatment or a problematic drug scene? – An interview study of patients’ and professionals’ views on the risks and benefits of liberalized opioid agonist treatment

**DOI:** 10.1186/s12954-025-01197-w

**Published:** 2025-04-04

**Authors:** Johan Nordgren, Torkel Richert

**Affiliations:** Malmö University, Malmo, Sweden

## Abstract

**Background:**

Opioid agonist treatment (OAT) is a highly effective treatment option for people with opioid use disorder. The potent medications used create dilemmas regarding low or high thresholds to access treatment, patient autonomy and regulations. OAT in Scania County in the south of Sweden has seen the implementation of regulatory changes resulting in liberalization through a patient choice model and increased access to treatment. In this setting, in which OAT has developed from high threshold to low threshold because of policy changes at both national and local levels, we aimed to analyze how patients and staff view the risks and benefits of OAT.

**Methods:**

We conducted qualitative interviews with 32 OAT patients and 15 OAT staff in Malmö, Sweden. We analyzed the data with a thematic analysis approach.

**Results:**

Patients reported that OAT helped them to “stabilize” their lives although many felt a sense of being locked into treatment, which acted as a barrier to normalization. A significant risk was being offered illicit drugs by patients and dealers when visiting the clinic. Patients who had enrolled in OAT before liberalization found current guidelines too lenient and expressed worry that persons being enrolled were too young. Staff viewed liberalization with some ambivalence, with a positive view of increased access to OAT, although they had worries about the enrollment of young patients and difficulties supporting patients with ongoing drug use. Staff also viewed the sale of drugs in and outside of clinics as a significant problem. Some staff viewed medicines as the most important aspect of OAT, while others positioned the social support as most important.

**Conclusions:**

Patient and staff perspectives were relatively congruent as they highlighted substantial risks regarding drug dealing at OAT clinics and were ambivalent toward the liberalization of OAT guidelines and the increased access to OAT. Liberalization impacted both patients and staff in their everyday lives and in professional practice, in a setting where OAT is both a desirable treatment and sometimes the basis of a problematic drug scene.

## Introduction

Opioid agonist treatment (OAT) with methadone or buprenorphine is the most effective way to reduce harms experienced by people with opioid dependence, with reductions in mortality, overdoses, law enforcement arrest and transmission of HIV and hepatitis C [21, 29]. However, OAT is more strictly regulated than other healthcare services due to the medical risks related to the medications and because of social stigma toward people who use drugs [4, 11]. Regulation pertains for example to ingestion of medication observed by clinic staff, drug testing of urine samples and policies on take-home doses [8, 31]. Strict regulations are commonly motivated by the risk that patients might sell their medications on the illicit drug market [15]. Regulations mean that although the treatment is effective in delivering positive health outcomes, some potential patients do not enroll in OAT. Some people who use opioids create their own OAT programs through buying methadone or buprenorphine illegally because of a will to cut back on heroin and to handle perceived barriers to OAT access [16]. These regulatory aspects of OAT can be categorized by reference to treatment accessibility barriers and treatment design barriers [10, 18] that encompass either low or high thresholds in OAT. Treatment accessibility barriers include highly selective and/or inflexible intake criteria, long waiting lists and the cost of treatment, while treatment design barriers include discharge policies, lack of individualization of treatment, lack of patient choice in medication and dose, choice of clinic, limited treatment, frequent screening for non-medical drug use and obligatory psychosocial treatment [18], i.e. factors that make it more difficult for patients to stay in treatment.

Although Sweden was one of the first countries in the world to initiate OAT with methadone, historically the treatment has been highly controversial and has been seen as being in opposition to the restrictive drug policy approach of Sweden [13]. Currently, OAT medications prescribed are methadone, buprenorphine (mono and naloxone combination) and long-acting injectable buprenorphine (LAIB). OAT in Sweden has been characterized by a high threshold model with strict admission criteria, and has had queues for up to two years to enroll in addition to patients being cut off from treatment if they broke rules or used illicit drugs. A notably harsh rule, now removed, previously banned discharged patients from OAT nationwide for three months [27]. These involuntary discharges have had a severe negative impact on OAT patients in terms of mortality, health, drug use, homelessness and criminality [9, 30].

OAT in Sweden has undergone significant changes during the last decade (see Table 1). OAT is regulated by national guidelines published by The National Board of Health and Welfare which state that OAT patients must be over 20 years old and must have been assessed by a physician as having an opioid dependency. Before the latest revision to the national guidelines in 2016, OAT could only formally be given to users of heroin, morphine and opium, but currently it is applicable for users of any opioid. The current guidelines state that continuous relapses by patients in treatment no longer represent a valid reason for discharge. The general tendency in OAT in Sweden has thus been a move from high threshold treatment to low threshold, meaning an increased acceptance among clinics and in the national guidelines that patients will not always be able to follow the intentions of the treatment providers [1]. In 2014, the Scania Regional Council introduced patient choice in OAT, allowing patients to select and change clinics. Both public and private clinics meeting accreditation criteria could now provide OAT. This regulatory change, driven by a right-wing political majority, aimed to boost competition and access in publicly funded health services [3]. Patient choice has facilitated the establishment of more private care providers that offer OAT in tandem with publicly financed clinics. In 2013, there were eight OAT clinics in Scania, while in 2024 there were 27. This enlargement in OAT providers in Scania resulted in a 53% increase in the number of patients between 2013 and 2017, from 992 to 1514 [1]. Both OAT patients and staff describe the effects of patient choice in terms of a sense of empowerment among patients [2, 3]. However, in 2024 the Scania Regional Council announced that the patient choice model would be terminated in 2025. This was mainly due to financial mismanagement in some private clinics enabled by the model of payment in which clinics earn money by increasing patient visits. In some cases patient visits were falsely registered to increase earnings. Another reason for the termination was that the development of long-acting injectable buprenorphine makes this payment model problematic (fewer clinic visits makes it difficult for private clinics to ensure economic stability).

**Table 1:** Overview of the development of OAT in Sweden

1960s: Introduction of methadone treatment in 1966 as part of a pilot program, reflecting growing awareness of opioid addiction as a public health issue.
1970s: Emphasis on abstinence-based treatments with methadone only available under strict criteria. Regulations were tightened significantly following concerns about diversion and misuse. Patients had to demonstrate long-term and severe opioid dependence, often requiring multiple failed detoxification attempts before qualifying for methadone treatment. Treatment was centralized, with only a few approved clinics allowed to prescribe and administer methadone. This limited access significantly compared to decentralized models in other countries.
1980–1990s: Limited expansion of substitution treatment, with methadone programs focusing on stringent eligibility requirements. Buprenorphine was introduced in the late 1990s but access remained restrictive.
2000s: Regulatory shift allowed for greater availability of substitution treatment. This marked a move towards harm reduction, increasing the number of treatment slots and the inclusion of more opioid-dependent individuals in methadone or buprenorphine programs.
2010s: Expansion of treatment continued, particularly through the “Patient Choice” reform in Scania County, which increased access to treatment by offering patients more provider options. Criticism of strict “zero tolerance” policies (e.g., discharging patients for non-compliance) led to more flexible approaches in some settings.
2020s: Greater emphasis on balancing accessibility with safety. Discussions about low-threshold programs for harm reduction emerged, addressing the challenges of diversion while maintaining patient retention in treatment. Substitution treatment, including both methadone and buprenorphine, had become a cornerstone of Swedish drug policy for opioid dependence, with increased focus on reducing high rates of opioid-related mortality. In 2024, Scania Regional Council decided to terminate the patient choice model.

For most people who use opioids, OAT has a stabilizing effect; however, OAT clinics may also become part of the subcultural drug scene in a specific setting. The treatment may be a way of severing or establishing connections to people involved in illegal drug use, drug selling and criminality. OAT clinics can thus become a scene on the illicit drug market where people who use drugs and patients meet, establish contact and offer sales of medicines or illicit drugs [12]). The dichotomy between OAT as both risky and enabling has implications for how patients and staff view the treatment, and how OAT clinics organize and try to shape the treatment modality [12].

The liberalized way OAT is delivered in Scania County, with lowered thresholds for treatment access and higher thresholds for treatment termination and a health care market for OAT has meant a shift in OAT delivery. The rationale for this study was to gain insight into how both patients and staff perceive the recent liberalization of opioid agonist treatment (OAT). This is particularly important given the scarcity of research that incorporates both patient and professional perspectives within a single local setting of OAT, as well as the inherent strengths and weaknesses of both low- and high-threshold OAT approaches. The aim of this study was to analyze the perspectives of OAT patients and staff regarding the main risks and benefits of the treatment in the setting of liberalized OAT in Scania County.

## Methods

We used a qualitative research design to achieve an in-depth view of the benefits and risks of OAT in Malmö, Sweden. We wanted to gain both patient and staff perspectives on the positive and negative aspects of OAT and thus conducted structured interviews with patients and semi-structured interviews with staff. In this section, we describe our methodological considerations relating to these two groups of interviewees.

### Patient interviews

We conducted 32 structured interviews with patients at two private OAT clinics as part of a larger survey of 231 people who use drugs in Malmö. We recruited patients at the OAT clinics by approaching people who were at the clinic to pick up their medicines or to meet with staff. The patient interviews were conducted by a female research assistant who also transcribed the interviews. The criteria for inclusion in the study were that the person was enrolled in OAT and spoke Swedish or English, and we employed a strategic purposive sampling approach in order to include a broad sample of patients in terms of age, gender, time in treatment, and social situation [23].

During the interviews, we collected both quantitative and qualitative data. The participants responded to a survey of 95 questions about everyday life, health, risks, social network, violence, safety and crime. As an addendum to this interview, 32 of the patients were asked additional questions specifically about their views of OAT. This concluding part of the structured interviews was transcribed and analyzed. The questions related to perceived risks and problems, as well as positive aspects of OAT. The answers were of varying lengths, but most interviewees provided rich descriptions of their experiences and views of treatment. Patients were reimbursed with a 200 SEK food retail store and pharmacy voucher for their participation.

### Patient characteristics

Demographic information on the 32 patients who participated in the interviews is presented in Table 2. The higher proportion of men in the sample reflects the generally higher proportion of men in OAT in Sweden, in which around one-third of patients are women [1]. Most patients were of Swedish ethnicity (*n* = 25) and the remaining patients were primarily from other European countries. Patients had an average length of drug use of almost 20 years and had been in treatment for an average of five years, although the total range was wide. Nineteen reported having a stable housing situation, such as living in their own apartment, while 12 reported an unstable situation such as living with friends or acquaintances or in temporary housing without a permanent contract. Two respondents reported that they worked full time, three reported no income, 15 were on social security benefits and the rest of the sample engaged in various activities to gain income, such as working odd jobs, dealing drugs or committing thefts (not shown in table).

**Table 2:** OAT patient characteristics (*n* = 32)

Gender	
Male	20
Female	12
Median age (range) [average]	41 (28–63) [43]
Median years of drug use (range) [average]	18 (6–40) [19.5]
Median years in OAT (range) [average]	5 (0–25) [6.2]
Housing situation	
Unstable	12
Stable	19

### Staff interviews

The authors conducted qualitative interviews with 15 OAT staff at six clinics in Malmö. The clinics included both regional and private service providers. We used a strategic purposive sampling strategy to interview a broad range of professionals within OAT, including nurses, doctors and social workers. Interviews were conducted face to face on the clinic premises and we used a semi-structured interview guide that included questions about daily work tasks, views of open drug scenes in Malmö, assessment of the situation of people who use drugs in the city, key issues regarding risks, dilemmas and positive aspects of OAT, cooperation with other services and views on Swedish drug policy. The interviews were recorded digitally and then transcribed by a professional transcriptionist. The interviews took around one hour to conduct, with a range between 55 and 110 min.

### Staff characteristics

Fifteen staff at six different OAT clinics participated in the interviews, with four staff from clinic 1, three from clinic 2 and two staff from the remaining four. Three of the clinics were operated by the public health care system and three were private companies. Staff were employed in positions including manager, physician, psychologist, nurse and social worker and they worked closely with patients in treatment, although with different roles. The participants were highly experienced, with a minimum of five years of working with drug addiction treatment, and the average age was 49 years. Staff demographics are shown in Table 3.

**Table 3:** Staff characteristics (*n* = 15)

Gender	
Female	13
Male	2
Median age (range) [average]	45 (30–66) [49]
Public clinic	7
Private clinic	8

### Coding and analysis

Our analytic strategy was based on thematic textual analysis [19]. We first analyzed the patient interviews by reading through all the transcriptions to get a general overview of the material in a process of familiarization with the data [28]. In the following step, we manually coded the patient interviews by assigning statements from the interviewees to a wide range of initial codes. These statements were added to codes such as “Being offered drugs”, “Life-long treatment”, “Relationship with staff” and “Young age as risk”. We then counted the number of statements in each category, and identified the most occurring ones. For example, “Being offered drugs” was the most frequent code in the patient interviews. However, other categories were less frequent but were still analytically interesting. As such, we employed a combination of inductive and deductive approaches in our coding and analysis [5]. The staff interviews were analyzed after the patient interviews, employing the same analytical strategy. Through a subsequent reflection of the identified codes, we identified central themes that emerged from the empirical material, with a focus on specific aspects of OAT treatment in the studied setting. In a final step, we chose quotations that represented the themes and translated those examples from Swedish to English.

### Research ethics

The study was approved by the Swedish Ethical Review Authority (Dnr. 2019–06509). We have anonymized the interviewees by assigning them numbers, and we have removed details that could identify an interviewee or his or her OAT clinic in the quotations.

### Findings

In the following two sections we first present the patients’ perspective and then the professionals’ perspective on the risks and benefits of liberalized OAT.

### Patient perspectives

#### Stabilization

Most patients reported that OAT enabled them to live “stable” lives and that it had improved their lives to a great extent, some even stating that the treatment had “saved” their lives. A particularly prevalent theme was that the treatment had allowed them to stop using illicit drugs. One female patient said that the most positive aspect of OAT was that: “I have continued to keep myself free of illegal drugs since I got my medicines” (Patient participant 6, female). Patients often described the medications as a key to their successful treatment and positive change of life, as in the following excerpt:

Well, I have a much, much better life today. I’ve been homeless for several years and I would never have made it here where I am today if not for the methadone, definitely not. And now I have had my own apartment for five months (Patient participant 26, female).

Although patients sometimes described positive support from the staff, they also ascribed great importance to the medication, as in this example of a patient who described the positive aspects of OAT for him:

I’m not going to say that it’s free opiates because that’s not what it’s about at all. It’s… I mean my true win win is that I get a stable everyday life, I get a more stable psyche. Not flat, but stable (Patient participant 14, male).

A significant part of stabilization is that the patients do not have to engage in the illegal economy to earn their upkeep and money for drugs. This kind of stabilization is exemplified in the following excerpt:

First of all, I have survived. That’s the major part and you get a stability and don’t have to chase and stress over fixing drugs and all that. So that part is gone completely. It becomes… for better or worse, it’s calmer, a calmer way of life (Patient participant 12, male).

Stabilization was also connected to mental health improvement during treatment:

It’s the routine among other things, and that, well I can avoid being illegal. And that it’s in a controlled form which means that I have my dose, I don’t get high and I don’t get wasted because it removes a lot of the craving and the other anxiety that I go through (Patient participant 31, male).

The rules and routines of the treatment were also seen as a part of stabilization by some patients. This related to having a place to go to, meeting staff that cared about them and knowing that positive drug tests could lead to more restrictions. For example, some patients said that they would refrain or try to refrain from using illicit drugs, since this could show up on urine tests, and if so would mean that they would have to come to the clinic daily to pick up their medicines and be further tested. Such rules are used by the clinics to make sure that the patients do not take medicinal risks. The patient in the following excerpt was not very concerned about using other drugs such as cannabis, but nonetheless thought about the risk of having to pick up his medicine daily if he tested positive on a urine sample:

I don’t know… I guess I have gotten a sense of control. I mean, I don’t want to come here and be positive. So it has made it possible for me to abstain. I mean, I don’t care really, if I become eager to smoke a joint I just do it. That’s the way it is. But I still have this thought that “Yeah, if I’m positive on that then I have to come here every day once again”. And that’s pretty boring (Patient participant 16, male).

There was great variation in what patients thought about the staff and rules. Some perceived the rules as too lenient while others found them too harsh. Most were satisfied with the support and treatment, but some reported powerlessness and being suspected and punished by staff. Overall, stability and routine were central words that the patients used to describe the positive aspects of OAT.

#### Being ‘locked in’

Although several interviewees reported that OAT provided them with a sense of stability in their lives, some described a sense of being “locked in” through their enrollment. The notion that OAT is a “life-long treatment” was prevalent in the interviews. Reflecting on her six years in OAT at the time of the interview, one patient wished that she had obtained more information about OAT and particularly about other treatment options. She had tried heroin only a few times and had a problematic use of buprenorphine before entering treatment, but a friend had told her that the rules had been changed so that she could receive OAT although her main problem was not heroin. She had been enrolled then, and described the situation as follows:

And then it was like… it was not “OK, you will get methadone now for this period of time and then we will try to taper you out”. It was… they said quite clearly to me that “Methadone is something you surely will have to take for the rest of your life”. And they just increased the dose you know. So then suddenly I sat there with much worse drug abuse than what I had before. Only more organized or how to say… (Patient participant 18, female).

She further elaborated to describe her sense of a lifeworld that revolved around the clinic and that this constituted a barrier to normalization:

It’s just a substitution and, yes, it’s almost… so you become less free when you go to a clinic. You have… it does something with you as a human being as well. It’s difficult to feel like a part of ordinary society and at the same time come here and feel like you are… like you are not capable as a human being (Patient participant 18, female).

Being locked in was also related to social stigma and association mainly with patients who experienced problems in treatment or who had been visibly scarred by several years of drug use problems, as in this excerpt:

What I find a little hard is getting down here among other addicts. People who may have been addicted for 60 years are still affected physically and mentally by the fact that you may become in a certain way. I don’t feel any belonging. It’s maybe a little hard for me. That I do not want to mix too much with other addicts and so on (Patient participant 32, male).

#### Being offered drugs at the clinic

The most central theme regarding risks of being enrolled in OAT was being offered the chance to buy drugs from dealers outside the clinic or from patients in treatment, with 21 of the 32 patients mentioning this as the main risk to them in treatment. The following quotation is a representative example of how dealing was discussed by the patients:

*Interviewer: What would you say are the main risks or problems regarding enrollment in OAT*,* as you experience them?*

I guess the risk is that you meet people who are active [in illegal drug use]. There are a lot of people who are active but still come here and try to sell to you outside. I don’t like that. But I feel quite stable so most of the time I say no. But sometimes I feel that… yeah… if you have a relapse or have a bad day and get that question… You often see that it’s the same persons [who try to sell]… if I have said no… and I kind of know most people here so I say to them… they know that I have had a long period of being drug-free and I will say no. Before, I kind of felt ashamed to say no, but nowadays it’s like “No I’m in the process of getting my kids back”, like that. But then the day after they might ask me again “Do you want this [drug]? Do you want this? “No!”. (Patient participant 6, female).

This quotation indicates a significant daily risk of relapsing for patients who must come to the clinics often to pick up their medicines. One interviewee described how she felt “stuck in this environment” and that other people offering to sell drugs at or outside of the clinic constituted a major problem for her and others:

It’s really difficult. I really had to… I know a lot of people who must come here and then go home immediately. Not chatting with anyone, not going on detours because if you have a bad day and people want to be nice and help you and they have stuff [to sell] then it’s really difficult to refrain (Patient participant 18, female).

Although being offered illicit drugs at OAT clinics is not a new phenomenon, the liberalized OAT landscape involving a more mixed patient group may create new risks and challenges for both patients and staff. Since this theme was also significant among staff, we return to it in Sect. 2.

#### Reactions to increased OAT access

Some patients were highly critical of a perceived increased access to OAT at the clinics. One factor behind the increased access is the establishment of patient choice for OAT in Scania County in 2014. A common perception, particularly among older patients, was that the clinics had begun to admit younger persons into treatment. In such comments, these patients defined OAT as a risky environment for younger and less experienced persons, as explained in the following quotation:

I mean, they give methadone. That’s ten times more dangerous than heroin. And they give it to brats who have smoked some oregano. Twenty-year-old guys and gals who they give methadone. Life’s up, it’s the last stop. You should have tried every single [other] treatment method there is. You should… I mean when I got methadone you had to have registered intravenous heroin abuse for two years. And now… it’s become such a… they open clinics like crazy. They don’t realize what they are doing (Patient participant 10, male).

As seen in the quote, this patient was also critical toward the opening of several private OAT clinics. The concern about the young age and limited opioid addiction of enrolled patients was also discussed in relation to the notion that OAT is a life-long treatment, and that young persons should try abstinence-based treatments first. The following patient felt strongly about admitting young patients into OAT:

There is a way too low age limit. Kids can stuff themselves with some tramadol and then they are in OAT and chew buprenorphine. And they have lowered it to the age of 18, I think it’s crazy. Insane that they use it. I think it should be 20, it [opioid use history] should be documented and it should be hard to get in here. It should not be like child’s play. /…/ If, for example, they receive methadone as an 18-year-old, they will never have a life. Sorry I get so excited, I just think it’s crazy (Patient participant 13, male).

Although the national OAT guidelines state that patients who enroll in OAT must be over 20 years of age, clinics have the option of admitting patients who are younger, provided that the patient has previously received other treatment services that have failed to improve his or her situation.

In the following section, we turn to the staff’s perspective on liberalized OAT.

### Staff perspectives

#### Access to OAT

Somewhat paradoxically, the liberalized national OAT guidelines and the increase of access to OAT in the Scania region was understood as a risk by staff, but also as a development of effective care for people who use opioids. The expansion of OAT in Scania has meant that more patients are enrolled in treatment. Some of the professionals reflected on this change. One professional described the changes in the national guidelines as significant: “I would say that before the threshold was high in and low out. Now it is low in and high out. So I think that it’s a big change” (Staff participant 15, female). Staff explained that some clinics previously had waiting lists for treatment that could be one or two years.

Another professional spoke in terms of a pendulum movement in OAT in relation to strict or loose admission criteria and possibilities to involuntarily dismiss patients:

/…/ it has gone from being actually difficult to get into OAT and it was super detailed, and you were kicked out for the tiniest thing. Which was not… that was not good. Sometimes I feel that maybe the pendulum has swung a bit too far in the other direction… it’s not so bad to have rules. They can be a support to cling onto (Staff participant 1, female).

The greater access to OAT was seen as positive in terms of being able to help more patients. On the other hand, professionals saw a risk that some patients could be enrolled despite not having severe opioid dependence. Loose regulations also meant that some patients continued a lifestyle connected to crime and illegal drug use or even worsened their situation during treatment: “Now they know that they can be enrolled here and still use other drugs on the side and never be dismissed because… we will accept it anyway so to speak” (Staff participant 13, female).

Another interviewee discussed the changed national guidelines about dismissal, mentioning that some patients may be enrolled for long periods even though they do not make progress in their treatment:

I think it’s a problem that you can feel that your hands are tied behind your back since we cannot dismiss patients anymore even if… I mean we might uphold a dependency for quite a number of years even though it does not benefit the patient or we might get nowhere. In a very few cases their situation might even become worse when they are enrolled (Staff participant 15, female).

This excerpt is similar to the view of the patient who claimed that her degree of dependency increased because of enrollment in OAT and indicates a shared perspective among some patients and staff. A significant theme in the staff interviews was the younger group of patients, which was perceived as having increased in size in recent years. Those in the group were described as younger males who mainly had a problematic use of tramadol or who engaged in polydrug use. One interviewee had experienced that these patients may have had a documented opioid dependency when they enrolled, but that staff over time could identify that their central problems did not concern opioids. Like several patients, staff members were concerned that young people with tramadol use were given access to OAT and given buprenorphine, as in this example:

When they put twenty-year-olds here who use tramadol and say “Well let’s give them buprenorphine instead” I think “They just switched to another drug”. And then they meet all the others [patients] here who are really hardened. I mean “Oh no” (Staff participant 10, female).

One interviewee had experienced an increase in the number of younger patients with less severe drug problems compared to those enrolled previously. In her initial meetings with potential patients in assessment meetings, she emphasized that there was a need for clear information about what OAT entails:

Since I’m the one they usually meet when they want to enroll, one of the things I bring up is that for most people this is a lifelong treatment. That you should perhaps not start this treatment with the intention that you will take it for a while and then quit, because that’s quite rare (Staff participant 7, female).

Staff and patients thus shared a view on younger persons increasingly accessing OAT as a problem in the new landscape of high availability of OAT in Scania.

#### Sale of drugs

The professionals were aware of the drug dealing that sometimes occurred at the clinics, in waiting rooms, outside the premises and in the public areas around the clinic premises. Like the patients, the staff identified the sale of drugs and being offered drugs as a significant risk associated with coming for treatment. Specifically, staff viewed this risk as higher for those who picked up their medicines every day and those who were not feeling well. One interviewee was hesitant to demand that a patient who had made progress toward a “drug-free” life must pick up her medicine on the weekends:

Because I know what it looks like here [at the OAT clinic] on Saturdays and Sundays, people hanging around, which people come here then. And since she is working so hard on being drug-free, I think it becomes a risk situation for her. And then we can perhaps help her so that she doesn’t have to come to the clinic (Staff participant 7, female).

Some staff commented that the patient choice had reduced the number of patients that pick up their medicines at pharmacies, since clinics are financially reimbursed for patient meetings at the clinic.

One staff member said that patients worry about coming to the clinic to pick up medicine because they are offered the chance to buy drugs from other people: “It’s not protected, it’s not a safe place to be in if they think that they are not going to use [illegal substances]” (Staff participant 5, female). There was some uncertainty about what to do to stop the dealing, but also concrete strategies taken by some clinics to stop or reduce it.

*Interviewer: The thing with open drug dealing or selling to each other or sharing*,* is that something you notice at the clinic or outside?*

Yes, we do sometimes but we are rather aggressive about that. Then we go out and disturb them and if we can’t handle it ourselves, we call the police and ask them to drive by the clinic a few times. It usually stops then (Staff participant 11, female).

Staff also had to handle gossip about selling and often felt that dealing was not included in their jurisdiction as staff employed in a medical clinic:

Sometimes we get information that “this guy is selling”. And what we really don’t like is when they sell medicine that they got from us, we get quite pissed off about that. But it’s not only that, other stuff is sold too. But it’s really difficult for us in the healthcare system to know what to do. We can… I mean we are not the police. So when that kind of information comes in we try to discuss it and check it (Staff participant 1, female).

Although staff would sometimes act against drug dealing outside of the clinics, some differentiated between what happens in the clinic waiting room and outside, as in the following quotation:

It’s almost impossible to come and visit an OAT clinic during times when there is an open reception, when a lot of people come, without being exposed to offers of drugs. /…/ That’s the way it is. And that’s a problem because indoors at the clinic we have the possibility to take some kind of action, but outside those doors, down the stairs, we don’t have any formal… It’s not the clinic that should uphold the law (Staff participant 9, male).

One professional explained that they had a few patients who wanted to cut down on their doses and stop medicating. To minimize the risks to those patients when coming to the clinic, they arranged matters so that they could visit after their drop-in hours, and some clinics had separate entrances for specific groups of patients considered stable. Some clinics adopted a strategy to employ security guards who at times would patrol outside the clinic in an attempt to limit dealing.

#### Medication versus rehabilitation

Like the patients, staff also viewed the medication as crucial for the stabilization and health of the patients. However, the staff also provided psychosocial support to their patients. In the following quotation, this staff member positioned the medicine as the central part of treatment, although she also found the social support important:

The medicine is the big thing, I guess that is the main part. /…/ Of course our main aim is the medicines… I mean it is substitution treatment, but I want to have the contact with the patients and you cooperate with other services such as the social services and housing services and all of that. And of course, the counselor is a key player who conducts supportive talks with them about what is happening in their lives and about their accomplishments, and their struggles too (Staff participant 10, female).

In the following quotation, a nurse staff member positions the medicine as a minor part of treatment, and instead emphasized the inherent importance of different types of psychosocial support:

*Interviewer: To what extent do you also aim at change or a long-term goal that they should change their lives*,* get a job and become integrated into society?*

We work on that daily. We are a kind of mother ship to them. I mean, the medicines are the small part. Then we push them to call the Public Employment Service or go to the social services. And we follow up on that. And a lot of things to do with housing. So we are more like social workers than nurses in a way (Staff participant 2, male).

Staff frequently noted that it was rare to see patients stop using the medicines. As one nurse who had worked with drug abuse treatment for 30 years said: “We must not forget that it is possible to zero patients. They are not many but it is possible. /…/ I have not done it at this clinic, but in my life I have zeroed no more than approximately five patients. It’s not a lot” (Staff participant 4, female). One clinic had decided to keep some patients who had tapered down entirely in enrollment, to be able to continue to provide psychosocial treatment, which otherwise would be the mandate of the social services to provide:

We are there for them for one year afterwards precisely because they should be given those psychosocial interventions if they wanted to continue. We saw that some patients did not want to quit the medicine because then they would have to stop being enrolled at the clinic. So we had to do it like that. And we have a few who come regularly and have no medicine (Staff participant 11, female).

The way OAT often was defined as a life-long treatment also raises the importance of reducing risks in the treatment and facilitating health and well-being for patients, for example by taking action against dealing at the premises.

## Discussion

In line with much previous research [6, 26], OAT patients defined the medications as key to achieving stabilization in their lives, which was related to a calmer way of life that did not center around alleviating abstinence, engagement with crime and the illegal economy. As such, the medication can be described as an important material resource in the patients’ lives [12]. The view of the medications was split among staff. Although most saw the medications as the central part of the treatment, and as crucial for patients’ stabilization and well-being, others viewed them as secondary to the psychosocial support. The staff’s focus on psychosocial support can partly be interpreted as this being perceived as decisive for the patients’ opportunity for positive change. It could also be understood against the background of the long history of criticism against OAT in Sweden, where focus on rehabilitation in conjunction with harm reduction has been a way of legitimizing a controversial form of treatment [7].

Although sometimes experienced as too rigid, the rules about demands on daily clinic visits and urine testing employed by the clinics toward patients with ongoing illicit drug use were described as an important part of the process toward stabilization by some patients. OAT patients in Canada describe giving or removing take-home doses by the MD as reward and punishment [25]. For some, this is a positive aspect of OAT in that it can create something to work toward, and it can be a reason not to use illicit drugs due to the chore of going back to picking up medicine every day, as brought up by some of the interviewees in the present study.

Dealing at or around the clinic premises was seen as the most pressing risk in treatment, especially concerning the risk of relapsing into illicit drug use for those patients who strived for abstinence and who frequently had to pick up their medicines at the clinic. This risk might partly be related to the patient choice reform in Scania, since the clinics are now reimbursed per patient visit to reduce the risk that patients with complex treatment needs would be turned down by the clinics [1]. This model might create an incentive for both private and public clinics to maximize daily or more frequent medicine pickups. A patient choice model based on a fee-for-service system allowing private clinics to bill the publicly financed healthcare system can lead to problematic results. This type of model was used in Canadian OAT from the mid-1990s until 2013 and became associated with claims of client coercion, pharmacy fraud and diversion of methadone, although dispensation fees received by pharmacies allowed for greater control over the treatment for OAT patients through their negotiation with pharmacies [22].

Drug dealing at the clinics was well-known by staff, who enacted a range of strategies to counter this, such as trying to disturb people selling, calling the police, and having separate entrances for patients at risk, who also might be allowed to visit the clinic after drop-in hours. Staff also had to handle gossip about patients who might be selling and attributed the responsibility of enforcing dealing to the police. A notable theme here is the way OAT clinics may develop into spaces that some patients and staff defined as open drug scenes. Patients described how they had to navigate around the physical space of the clinic, with a temporal dimension for some patients who wanted to avoid people they knew were using illicit drugs or who offered to sell their prescribed medicines to them. This risk is also described in a study of OAT in Denmark, which found that patients described worries about drug dealing, being threatened, robbed, temptations of using illicit drugs and encountering persons they wanted to avoid [12].

The recently increased access to OAT in Scania County was described as a particular risk to young persons, which can be interpreted as showing care toward young people who experience problems with opioid use but who are still not as far into their drug-using careers. Patients and staff shared a similar perspective on the risks of enrollment for young persons with a shared worry about them developing more severe addiction problems and hardening into a criminal lifestyle by way of socially interacting with patients who continue to engage in the illegal drug economy. The issue of the age of enrolled OAT patients among the interviewees can be interpreted as concern for the young, but also as a symbolic boundary between “us and them”. This boundary seems to imply a definition of “real drug addicts” versus those who are not yet “done” with their problematic illicit drug use. Additionally, older patients who were enrolled under the strict high threshold guidelines felt that they had to fight to get into treatment, while those enrolled under the new guidelines were admitted too easily. Concerns by both patients and staff regarding age and degree of dependency problems in new patients highlight peer group dynamics and social norms. Social norms are especially apparent in the patients’ criticism of the recent changes to accessibility criteria in OAT in Scania. Some patients had experienced strict admission criteria when they enrolled before the liberalization of guidelines, and they were both upset and worried about the development. In this worry there is a sense of compassion for young persons who they argued should not have to risk being locked into a life-long treatment. Age emerges as an important factor in assessments of OAT eligibility, both for staff and patients, in tandem with implicit assessments of the extent of drug use or degree of dependency. A study of British patients’ perspectives on an “ideal” methadone program found that 43% of the sample suggested a minimum age of enrollment of 16 years, while 30% viewed an age limit to be unnecessary [17]. In the setting of liberalized OAT in Sweden, the issue of age at enrollment, which was previously a central point of concern due to the controversy of OAT [14], seems to be reappearing.

Staff also discussed the changes in guidelines with a focus on difficulties in dismissing patients who did not follow the rules or behaved in a threatening manner, whose situation worsened after enrollment or who engaged in continued illicit drug use. Staff acknowledged the development of OAT in Sweden toward a low-threshold approach that was described as “low in, high out”, particularly in relation to the previous strict national guidelines as well as local practices at the clinics. The tendency toward a more low-threshold approach in Swedish OAT has meant that more patients who previously would have been dismissed from treatment now stay on in treatment. This is positive for the patients who now can remain in treatment but can be a problem for some patients who do not want to be involved in illegal drug use or criminality. This is similar to the development in Denmark, where a liberalization of OAT guidelines and local practices has reduced the distance between the illicit drug scene and OAT clinics, with the result that they are seen as risky places by many patients [12]. Some staff in our study also said that the expanded patient group could negatively affect their work environment in the form of increased conflicts or threats.

The history of abstinence-only drug policies in Swedish drug policy and in local and national drug treatment settings affects views about OAT today, which is evident for example in some patients’ critical views of current enrollment guidelines and practices in relation to when they were enrolled. This also seems to be a reaction toward the liberalization of guidelines and local rules in OAT treatment. It is clear that the political decision to establish patient choice in OAT increased the coverage and availability of OAT in Scania County and in Malmö [1]. Both patients and staff viewed these changes with a degree of ambivalence. Although access increased, many patients felt that OAT operating regulations are still strict, such as urine tests and supervised oral medicine administration, and report a sense of being locked into treatment. This is in line with previous research that highlights patients’ concerns regarding inflexibility related to the clinic atmosphere and issues concerning frequency of clinic attendance, and opening hours [20].

This study of a changing OAT policy landscape highlights some of the dilemmas of OAT. Including a wider patient population reduces risks for the most vulnerable people who use drugs and could also reduce the demand for heroin and illegal drugs on the street [15]. At the same time, it can mean increased difficulties for staff involved in treatment, change the environment in and around the clinics, and increase risks for patients who want to stay away from illegal drugs. The study points to the need to continue to make OAT a more inclusive and enabling form of treatment while developing strategies to mitigate risks such as drug dealing, diversion of medications, hardening of younger people with less extensive addiction, and lock-in effects related to both medication side effects and processing rules and requirements.

Differentiation of several clinics with different target groups and orientations could be a way to reduce risks. The recent introduction of long-acting injectable buprenorphine in OAT in Sweden will potentially have a positive impact on OAT clinics as drug scenes, since patients need to visit the clinics less frequently, i.e., weekly or monthly [24]. However, the introduction of LAIB clashed with the pay-per-visit model in the patient choice reform, which was one argument for terminating the model in Scania. As such, the Scanian model of patient choice reform in OAT is an example in which privatization and liberalization of treatment with a pay-per-visit model became problematic in the view of some patients and staff, and might serve as an interesting example for policymakers. Further research might analyse how the termination of the patient choice model might have negative consequences for patients in terms of increased worries about OAT access and how the transition of patients to new clinics is carried out.

### Limitations

Because of our strategy of recruiting patients at the clinics, we have reached an older group of patients and patients who might come daily to pick up their medicine, which may have resulted in a higher proportion of patients who experience problems with the treatment, or who recently had a relapse. Since we focused our interviews on patients at two OAT clinics, and interviewed staff at six different clinics, the staff interviews are broader and patients’ views on their treatment are mostly limited to the two clinics, although some may have had experiences from other clinics, considering the patient choice regulations allowing patients to switch clinics.

## Data Availability

The datasets generated during and/or analyzed during the current study are not publicly available due to pseudo anonymity of research participants, but are available from the corresponding author on reasonable request.
